# Book Reviews

**Published:** 1818-10

**Authors:** 


### CRITICAL ANALYSIS
### *OF RECENT PUBLICATIONS,*
IN THE
### DIFFERENT BRANCHES OF PHYSIC, SURGERY, &c.

---

*Transactions of the Association of the Fellows and Licentiates of the King's and Queen's College of Physicians in Ireland.* Vol. I, 8vo. pp. 494. Dublin.

WE briefly noticed this very valuable work in our last Retrospect, and have much regretted that we have not, before this, been able to make our readers more particularly acquainted with its interesting contents.

"The science and the practice of physic (say the editors in the Preface,) have owed so much of their advancement, during the last half century, to public Journals, and the Transactions of various Learned Societies, that it were now superfluous to conciliate the indulgence of the profession towards a new work of this miscellaneous kind. The spirit of research is obviously promoted by whatever tends to open fresh avenues, or multiply opportunities, of communication among the inquisitive and well-informed. Detached fragments of knowledge are thus brought to light, which might otherwise have been lost or overlaid by extraneous matter; and a common sympathy and co-operation obtained in the labours of professional improvement.

"In all practical sciences, founded on experiment and analysis, facts and testimonies, however miscellaneous or insulated, have a relative as well as separately intrinsic value. In the latter view, they serve as marks or guides for the breaking up of new ground, to enlarge the field of inquiry; in the former, they are subservient to the higher purposes of scientific truth and arrangement. For, the enlightened practitioner, (to borrow the language of an eminent philosopher,) 'distrusting conclusions which rests upon this or that individual case, is anxious, by combining those of an immense multitude, to separate accidental conjunctions from established connexions, and to ascertain those laws of the human frame which rest on the universal experience of mankind.'

"In the year 1816, an Association of Fellows and Licentiates of the College of Physicians in Ireland was instituted, to hold monthly meetings for the communication of medical and philosophical intelligence. Hospital reports, morbid histories, and other original papers, are read at these meetings; and the volume of Transactions now offered to the profession consists of a series of such communications, as their respective authors have consented to render public."

This volume commences with the history of a *Case of Ulceration and Rupture of the Stomach;* by Dr. Crampton.—The subject of it was a lady, æt. 29, of a sallow complexion, and spare habit. At three o'clock in the afternoon, she was seized with a sudden spasm, as she termed it, in the stomach, which made her apprehensive of immediate dissolution. She had long been subject to pains in her stomach and in both sides; but they generally gave way to medical treatment in a few days. At five o'clock, when Dr. C. saw her, she suffered agonising pains in the whole abdomen; they seemed to originate from the pit of the stomach, as from a centre, and shot to both hypochondres, to the lower part of the belly, back, and shoulders. The abdomen felt hard, but not tumid, the abdominal muscles being strongly contracted; the pulse was not hurried, and the tongue was clean. At seven o'clock, her pulse was 100; her skin hot, and the pain still more urgent. At ten o'clock, the pulse was 120, much smaller, the breathing hurried; and, shortly after this, the capillary circulation began to fail. At twelve o'clock, the pulse could scarcely be felt; the head, feet, and knees, were cold; the face livid, and the breathing more embarrassed; she moaned without ceasing; her respiration gradually became shorter; her extremities colder; her stomach never rejecting either drink or medicine; and, retaining her senses and intellect to the last, she expired in agony at three o'clock in the morning. General and local blood-letting, fomentations, purgatives, glysters, blisters, and the warm-bath, had all been employed, and the use of them carried to the greatest extent.

"*Dissection.*—On opening the abdomen, the stomach was found flaccid and empty, it was pursed up and divided in some measure into two cavities, by a band of contraction, which ran perpendicularly across its anterior surface; this contraction was occasioned by schirrous indurations which followed the same direction. The contents of the stomach had escaped into the cavity of the abdomen, through a round aperture, situated at the anterior surface at the union of the cardiac and pyloric portions; oatmeal and castor-oil were recognised amongst the other contents of the abdominal cavity.

"The perforation of the stomach was perfectly circular, about the size of a large pea, and appeared to be the result of an ulcer on the mucous surface, which had gradually penetrated the other coats. The ulcer was hollow, nearly the size of a shilling, and had the appearance as if it had been made with caustic, with the orifice in its centre.

"There were extensive and recent signs of inflammation throughout the whole peritoneum, investing the intestines, which appeared as if they were injected; likewise exudations of lymph, which connected the convolutions of the intestines to each other by adhesion, were observed.—The liver and spleen appeared shrunk and flaccid, not indurated; the gall-bladder contained some yellow bile; the urinary bladder was empty and contracted."

Dr. Crampton, in his observations on the above case, notices how quickly the extraneous substances which had escaped into the cavity of the peritoneum excited inflammation; and how much sooner the fatal termination occurred than in ordinary cases of abdominal inflammation, although no mortification of any organ or of any texture had taken place. The same thing may be observed in all the cases of rupture of the intestines which have been related; severe and general peritoneal inflammation has ensued, which has terminated fatally in a few hours.

Miss H. the subject of the above case, had for three years been seldom altogether free from pain in the stomach;—she received much relief at different times, by applying leeches to the region of the stomach, by mild aperients, and a restricted diet;—the measures advised by Dr. Crampton. But she had been much accustomed to try to relieve herself by lavender drops and other cordials, to strengthen her stomach by tonics and hot wine,—a practice to which she had been injudiciously advised. Dr. C. observes, that ulceration of the stomach may be a disease of more frequent occurrence than we imagine; and he very judiciously remarks, that much injury is often done in obscure diseases of this organ by the exhibition of stimulating medicines, which, in cases of altered organization, must tend to lead the disease to a fatal termination.

*Cases of Tumours within the Abdomen;* by WILLIAM STOKER, M. D.

The subject of the first case was a gentleman, twenty-four years of age. It is difficult to form a decisive opinion on the nature of this case; the account of the dissection is so very unsatisfactory. It however appears, that an abscess had formed in what, from the description given, appears to have been a preternatural cyst. This cyst extended from the left lobe of the liver, superiorly to the diaphragm; laterally to the superior part of the stomach and spleen, and inferiorly to the upper part of the small intestines; so that through its intervention and adhesion all those parts were united; the liver was much enlarged, and of a pale colour, and when cut into was of a much firmer consistence than natural, but was not the seat of any part of the abscess. This cavity communicated by ulcerated openings with the stomach and the lungs. The opening into the stomach appeared recent, that through the diaphragm of long existence, as its edges were cicatrized.

The disease appeared to commence with symptoms of inflammation in the abdomen; three weeks after which, a copious evacuation of blood and pus by stool took place: in another fortnight, an evacuation of the same kind occurred from the lungs, which continued to occur occasionally for two years; at the end of this time the patient had regained a tolerably good state of health. One evening he felt oppressed at his stomach, and took an emetic; this did not produce vomiting, but was followed by head-ache, delirium, and a sensation of suffocation. In a few hours he died. The rupture of the stomach, no doubt, occurred soon after taking the emetic, and was the immediate cause of death.

Two cases are next related, which appear to have been diseases of the ovaria; in one of them the fluctuation of a fluid was perceptible. These were cured by the use of bandages, inunction with mercurial ointment and camphorated oil, and digitalis.—The two following cases are particularly interesting:

"M. M——, thirty-four years of age, very plethoric, consulted me in the year 1803, on account of dyspnœa, cough, febrile exacerbation, palpitation of the heart, flushing of the face, and incubus; his pulse full and rebounding; he had some time previously been under Mr. Richard's care, on account of a tumour, which, on examination, I found to be an aneurism of the gluteal artery, on its passing outside the pelvis, and had acquired the size of an orange. Besides other remedies, Mr. Richard's applied a compress over the aneurism, which had been kept on several weeks, and had prevented its further extension, but without any diminution of it. As the patient refused my request to have a consultation, I directed him to continue the pressure, as ordered by Mr. Richards; and, besides a light vegetable diet, and gentle laxatives, I directed digitalis in pills in such quantity as produced a decided effect in moderating the great and general vascular action, and consequently the symptoms connected with it. The size of the tumour soon began to diminish very sensibly under the continued pressure, and in less than fourteen days was entirely removed; nor has there been since any return of either the general or local complaints."

"Mr. S——, twenty-five years of age, was affected in the spring of 1808, with a severe attack of pneumonia, on account of which, besides other medicines, I directed V. S. freely. In about three weeks after convalescence, he called on me to shew a tumour, which formed in the flexure of the left arm, and which, on examining it, I found to be an aneurism of the brachial artery, with strong pulsation,—pulse at the wrist of the right arm very full and quick. He had become even more plethoric than he was before his late attack of pneumonia: the accompanying symptoms were so very unpromising, that my friend, Mr. Crampton, was consulted on the same day: and, in consultation with him, it was determined to make trial of external compression, whilst digitalis should be administered, as in the case of Mr. M. in which I believed it had been beneficial.

"The effect of the powders of digitalis in this case, given to the quantity at first two grains daily, and gradually increased to four grains per diem, was apparent in diminishing the violence and frequency of the arterial action; and the pressure being continued, and adapted by Mr. C. to the circumstances of the case, the aneurismal sack was entirely obliterated in less than fourteen days. I have very lately learned that this gentleman was carried off by pectoral disease, in the course of last spring, and that on examination of the body, after death, by Dr. Harty, the heart and aorta were found much enlarged."

*Case of Suppuration of the Liver;* by John O'Brien, M.D.

This occurred in a young woman aged eighteen, whose diet for some time had been extremely spare; but, on going to a place of service, she made a very hearty dinner; after which, she said she felt a hard lump in her stomach, which continued unceasingly; eight days after this she was admitted into the Fever Hospital. She then had "an enormous swelling and tension of the whole epigastric region, with pain in the right side, much aggravated by inspiration or pressure. The tension, in some degree, affected the whole abdomen; the pain occasionally extended to the scapula; and she complained of weakness and numbness of the right arm. The tumour presented no resistance to the touch, nor appearance of hardness. Her countenance was pale and anxious; pulse small and feeble, about one hundred and twenty;—she had no vomiting, nor yellow colour of the skin." A strong purgative, and a blister to the side, were first employed; and afterwards, large doses of calomel, joined with opium, were given every night. No abatement in the size of the tumour having taken place at the end of a week, it was considered advisable to open the abscess. About four pints of a dark brown or coffee-coloured fluid, occasionally mixed with pus, were drawn off.—On the sixth day after the operation, the patient died. "She languished for the interval without much change; affected, however, with rigors and hectic fever, with very laborious respiration."

On *dissection*, the intestines were found free from disease; the stomach, about the middle of its lesser arch, was inflamed to the extent of about a crown-piece, by which it adhered to the base of the abscess. The abscess involved about two-thirds of the entire substance of the liver, and was completely bounded by adhesions.

### *Dissections of Two Habitual Drunkards;* by SAMUEL BLACK, M. D.

The author commences with a reflection on the importance of restraining the disposition we have to generalize facts and establish *principles;* a caution necessary in scientifical enquiries, but more particularly so in pathological investigations. This reflection was excited by the very different effects, apparently arising from the same cause, witnessed in the bodies of two men. One of them was a journeyman baker, aged twenty-seven, who had been accustomed for the last two or three years to indulge in long protracted fits of drinking. He had been liable, for a considerable length of time, to a variety of stomach complaints, such as acidity, flatulence, vomiting, &c. His feet and legs had lately began to swell, and he had been twice tapped within the last month. The only very striking morbid appearance was in the liver, which was not one-half its natural size; its substance felt perfectly hard and rigid, and was full of hard tubercles of a dark brown colour, about the size of a garden pea: none of them shewed any tendency to suppuration. The gall-bladder had a shrunk and shrivelled appearance, and contained scarcely any bile. The pylorus appeared rather thick and indurated.

The other was a man, aged sixty-three. He had been for fifteen years an habitual drunkard. Within the last eighteen months he became affected with a variety of stomach complaints, such as loss of appetite, nausea, acidity, flatulence, obstinate costiveness, pyrosis, and vomiting. These continued to increase: for six months scarcely any solid food could be taken; and the little that was swallowed was almost immediately rejected by vomiting.—On examination of the body after death, a considerable serous effusion into the cavity of the abdomen was observed. The liver, especially its left lobe, was considerably enlarged, and its whole substance abounded with tubercles of about the size of a hazel-nut: when cut into, they appeared of a yellowish colour and a granulated texture, but did not contain any pus. The gall-bladder was pale, small, and quite empty. The spleen appeared more rigid than natural, and the entire viscus was not one-half the usual size; but its structure did not appear materially altered. The stomach was so small and contracted, that its cavity would not have contained a turkey's egg. The coats of it were thickened, and indurated in an extraordinary manner. Their original organization seemed entirely obliterated, and they had formed a solid homogeneous substance, which in some places was half an inch thick, in others three quarters of an inch. This substance, Dr. B. observes, in structure and appearance, resembled cartilage softened, more than any thing else he could compare it to. The pylorus, with difficulty, admitted the end of the little finger; the interior surface of the stomach abounded with several appearances, to which, the author observes, for want of a better term, he gives the name of fungous excrescences: some of them were nearly as broad as a shilling, and from their surface there oozed a dirty brownish fluid.

*Case of Gouty Affection;* by SAMUEL BLACK, M.D.

A gentleman, aged thirty-three, who had suffered two paroxysms of gout in the feet, became affected with pain across the lower part of the back; this, after it had existed for three months, was temporarily relieved by an attack of gout in the feet; it, however, recurred when that subsided, and a moveable tumour soon after became apparent in the cavity of the abdomen. The patient's health then began to fall off, his digestion being much impaired; and, in about two months, he died. On *dissection*, a tumour was discovered nearly as large as a man's head, which appeared originally to have sprung from the mesentery, but was now intimately connected by adhesion with all the circumjacent parts. It was of a whitish colour, soft and pulpy; and, on a general and distant inspection, exhibited much the appearance of brain. On cutting into it, the left kidney was found imbedded in its centre; this viscus was not altered in structure. The spleen was of the natural size; the liver considerably enlarged. The caput cœcum coli was much enlarged, and the enlargement consisted in a great quantity of pulpy substance, the same in appearance as that which constituted the mass of the tumour already described.

*A Case which shews the Influence of Hepatic Disease over the Functions of the Uterus;* by THOMAS MILLS, M.D.

The patient was a young married lady, who had suffered for some time from indigestion and an unhealthy state of the bowels, which had become attended with fluor albus. These complaints were removed by medicine; and she shortly after, became pregnant.

*A Remarkable Case of Hydrocephalus Internus, in an Adult, in which much Benefit resulted from the Administration of Dover's Powder;* by WILLIAM BROOKE, M. D. M. R. I. A. President of the Association, &c.

It is long since we experienced so much pain and regret, in the perusal of a history of disease, as we have done on reflecting on that before us. We first intended to pass it over unnoticed, trusting that there are few medical practitioners of the present day so deficient in pathological knowledge as to be led to imitate the practice here proposed; but further reflection made us consider that it were better many of our readers should think our remarks superfluous, than that only one should be induced to follow error, which he may so easily be made to avoid. In extenuation of the pathology and mode of practice here exhibited, we may remark, that the case occurred sixteen years since: this may, in some degree, serve as an excuse for the measures then adopted; but we are utterly at a loss to imagine, how the President of the Association of the College of Physicians of Dublin could make such an history public at the present period. For, observe, he has not recorded it as an instance of his errors and their fatal consequences, but to shew the beneficial effects of the measures adopted.

A young man, aged twenty, was attacked for two successive days with nausea, vomiting, and rigor, followed by a hot fit, and accompanied with very acute pain across the forehead, particularly severe in the temples. The eyes were suffused, very impatient of light; the pulse ninety; skin hot and dry; tongue white. He took some antimonial powder and opening medicine; and *two leeches* were applied to his temples.

On the following day (Sept. 12) he was worse; he had no sleep the preceding night; his eyes were much suffused, the pupils contracted, with intolerant of light; and he was much distressed with vomiting. A blister was applied to his head.—The following day, some opening medicine was given him. On the 14th, the pain of the head was excessive, attended with furious delirium: more opening medicine.

Our readers will have some difficulty in believing, that we have noticed all the remedial measures that were employed; they will hardly think it possible, that a young man could exhibit all the symptoms of *acute inflammation of the brain;* and his physician merely direct a little antimonial powder, two leeches, a blister, and some opening medicine, during the first four days: but let them withhold their admiration for a while, and attend to the observations of Dr. Brooke.

"Finding myself baffled in every attempt I had made to relieve him, and aware of the fatality of the disease, I begged for assistance, and Dr. Percival was requested to meet me the next day. In my evening visit, however, I found him in a state as truly melancholy and deplorable as can well be conceived, labouring under the extremity of pain, without a hope of recovery, or even mitigation of his sufferings. *A case of severe pain in the head, in which I had lately given opium with success,* LUCKILY *at the moment, occurred to my recollection; and, conceiving that the present exigency warranted any attempt,* I ordered him a draught composed of forty drops of vitriolic æther, and ten of the tincture of opium, in some mint-water."

We believe few persons will peruse the above passage without wondering how such a mode of reasoning could be employed by the president of a highly respectable College of Physicians.

A man of the meanest capacity, reflecting on such an assemblage of symptoms, would immediately see the necessity of using the lancet; but a pre-conceived opinion respecting the nature of the disease seems to have directed the mode of treatment, rather than the indication of the symptoms. The young man, it appears, had for some years a notion that he should die of water in the head; and the Doctor was very ready to adopt the same, for he called the most clear and indubitable case of acute inflammation of the brain, *hydrocephalus;* and the mode of treatment he had recourse to, was such as would have been employed for the cure of that disease.

For six days longer, opium, in the form of Dover's powder, was the only medicine employed; after that time, mercurial inunction was used: on about the twentieth day from the first attack, he died. On examining the brain after death, its blood vessels were found very turgid, and the lateral ventricles contained about eight ounces of serum.

*On the use of Oxygen Gas in Angina Pectoris;* by ROBERT REID, M.D.

It was exhibited to a patient who had for several years been subject to paroxysms of pectoral angina, which had usually been relieved by blood-letting; but this had lately failed in its efficacy, and the patient had fallen into a state of great debility. The duration of the paroxysm was immediately interrupted on his inhaling about a quart of the gas, and the recurrence of it prevented by the same means; but the patient shortly afterwards died from the previous effects of the disease. It does not appear that the body was examined.

*On the Nature and Treatment of Tetanus;* by the same.

We shall merely give a general account of this paper, as we shall be obliged to consider the opinions of Dr. Reid more fully, and compare them with those of others, when we take up a work, now lying on our table, which he has published on this subject. Dr. Reid, from reflecting on the phenomena which accompany this disease, was led to suppose, that the cause of it existed in the nervous system of the spine; and he mentions a case, in which examination after death evinced the correctness of this opinion. A beggar-boy, who had been long exposed to every vicissitude of season, had his foot burned by sleeping in a lime-kiln. Four days afterwards, tetanus came on, and proved fatal in thirty-six hours. On dissection, the viscera of the abdomen and thorax appeared perfectly natural, and there was no morbid appearance in any of the muscular parts. The brain appeared healthy in every respect, except some increased vascularity in the investing membranes. In the spinal canal, there was a considerable effusion of blood into the cellular texture surrounding the nervous mass; the latter appeared highly vascular, and the vessels remarkably tortuous; corresponding in situation to the ninth and tenth dorsal vertebræ, there appeared a whitish substance, very nearly resembling the medullary matter, effused between the arachnoid coat and pia mater, occupying the space of about an inch and a half, and covering about half the circumference of the nervous mass: on breaking the membrane enclosing it, it could be wiped off, and there could not be the slightest rupture discovered in the pia mater or any of its vessels. The only unusual appearance in the nervous mass itself was a deeper tinge than natural in its cortical and medullary parts.

Dr. Reid states that he has in several other instances discovered serous or sanguineous effusion, and other marks of inflammation of the investing membranes of the spinal cord.

The treatment recommended by Dr. Reid is to apply a blister to the whole length of the spine, and to act on the bowels by powerful cathartics. We should then endeavour to produce copious perspiration over the whole surface of the body, by means of active sudorifics.

*Three Cases of Melaena, in which the most decided good effects appear to have been produced by the exhibition of the Oil of Turpentine;* by W. Brooke, M.D.

A gentleman, about fifty years of age, who had been accustomed to have his mornings occupied by business, and who spent his evenings among his friends, came to Dublin in October, a stranger, and without any occupation that interested or amused him. He had hitherto been healthy, but he now became dyspeptic. Purgatives produced large evacuations of a black tar-like matter, with considerable relief. He went on in this way until the end of December; when light tonics and the blue-pill were given him. He took those medicines until March, and for some time had apparently much improved ; when he became worse, passing stools, appearing to consist entirely of thin tar, and perfectly inodorous. He was much sunk ; the lips, tongue, and gums, were quite white, and the countenance singularly palid. Oil of turpentine was then given him. On the second day after taking it the stools became natural, and no appearance of the pitchy matter could afterwards be detected. He, however, still continued to suffer from indigestion, and died in July following.

Mr. T. B. aged twenty-two, had been subject to attacks of pain in stomach and bowels, frequently succeeded by vomiting, accompanied with costiveness, loss of appetite, and general debility. During an unusually severe attack of this kind, he passed large quantities of liquid, black, inodorous, matter, which continued for several days, producing great exhaustion. Twenty drops of oil of turpentine in cinnamon-water were exhibited three times a-day. After taking two draughts, he passed a motion chiefly composed of natural-looking fæces. He continued the use of the turpentine a few days, and quickly regained his health.

Miss M. aged forty, was suddenly seized, after breakfast, with vertigo and faintness; she became sick, and vomited a large quantity of matter, which appeared to her friends to be black blood. A few hours after this, she complained of a distressing sensation about her head, with occasional inclination to vomit, and great general weakness. The pulse was quick, skin warm, and her bowels had not been open for three days. Opening medicine produced several copious stools, "composed, in a great degree, of grumous blood;" she soon after again vomited a large quantity of "black, bloody matter." Her countenance became sunk and much changed, and her lips and nails quite white. She complained of no pain; the bowels were soft and flat; pressure on the abdomen produced no uneasiness. Twenty-five drops of the oil of turpentine were directed to be given every sixth hour. During the night she had four stools, composed of a tar-like matter. The turpentine was continued: the following day she had had no stool, but felt much better. Saline opening medicine was given, which produced some stools, black and inodorous. From this time they became natural, and Miss H. soon recovered her health.

*On Dropsy and Apoplexy;* by WILLIAM STOKER, M. D.

This relates to a case in which anasarca accompanied inflammation of the lungs, and a general plethoric state of the system, that was removed by blood-letting. Such cases are not uncommon; but, from not having recourse to bleeding for fear of inducing hydrothorax, they have frequently terminated fatally. It will be found, in the greater number of instances, as in the case here related by Dr. Stoker, that the dropsy will disappear almost immediately after blood-letting; but, if that the only remedy be neglected, the patient will either die from the more immediate effects of pulmonic inflammation, or from serous effusion into the cavity of the chest.

*A Case of Chronic Rheumatic Inflammation, successfully treated by Bandages;* by RICHARD GRATTAN, M.D.

Many pages in the late numbers of our Journal have been devoted to the reception of histories of the extraordinary effects of pressure in the cure of diseases, and we feel much pleasure in adding to the list so decisive an instance of its salutary powers as the case here related by Dr. Grattan furnishes us with.

Mrs. C. aged 35, a few weeks after having been exposed to wet and cold in an open car, experienced a loss of power in her feet and legs, attended with a sensation of numbness. This continued to increase, until she was unable to support herself. Her feet afterwards became excessively painful, and the joints of the ankles and toes were much swelled. All the usual remedies were tried, without benefit. The feet at length became distorted; being permanently drawn inwards and downwards, so as to completely hide the inner ankle. The slightest touch produced exquisite pain, and the pressure of the bed-clothes was intolerable. Dr. Grattan was induced to have recourse to the means so highly recommended by Dr. Balfour. The feet were bathed in salt and water, and enveloped in bandages; the result was so favourable, that in a short time Mrs. C. was enabled to attend to her domestic duties with as much activity as at any former period.

*Case of Hydrocephalic Fever;* by John Crampton, M.D.

We are sorry to see an interesting and well related case, designated by a term so unmeaning, and likely to mislead the inconsiderate. It appears to us, from the symptoms detailed by Dr. C. to have been a case of common inflammation of the brain, which, it is very probable, might have terminated in hydrocephalus, had it not been judiciously treated. We have too frequent occasion to lament the evils arising from naming a disease from some one of its consequences; and men, whose opinions are entitled to attention, should not encourage what is so deleterious by its influence over the minds of unreflecting persons. Dr. C.'s patient was a boy, twelve years of age. Leeches, in considerable numbers, were applied to the temples, and James's powder, digitalis, and calomel, copiously exhibited. He finally recovered.

*Case of Abscess in the Liver;* by Edward Geoghegan, Member of the Royal College of Surgeons in Ireland, and Surgeon to the Dublin General Dispensary.

A man, twenty-four years of age, had laboured a fortnight under great pain in the region of the liver. He had been twice copiously bled, freely purged, and blisters had been repeatedly applied. After this, he was seen by Mr. G. who discovered a deep-seated fluctuation; calomel and salts were directed for him; and it was proposed to him to have the matter evacuated,—this was not agreed to until a fatal termination was speedily expected. About six pints of matter, mixed with bile, were discharged; immediate relief succeeded, and gradual amendment during twenty-three days, when diarrhœa took place, and proved fatal in a few days.

*Case of complicated Dropsy, and Dissection, with Observations;* by P. E. M'Loghlin, M.D. M.R. I.A.

The subject of this case was a woman, aged fifty-four; she suffered from cough, dyspepsia, dysuria, and the usual symptoms of dropsy. Sixteen quarts of serum were found in the cavity of the abdomen after death. The stomach and whole intestinal canal were remarkably contracted; and, from the last sigmoid flexure of the colon, through the whole course of the rectum, the canal was nearly obliterated. The intestines had also formed adhesions, throughout their whole extent, with the parietes of the abdomen. The liver was not larger than an ox's kidney; the gall-bladder contained healthy bile; the spleen was of a remarkably small size; the bladder was so small that it could not contain more than two ounces; the ureters were as small as threads, and became evanescent in the adhesions; the uterus was enlarged to the diameter of about four inches; the left ovarium contained about three pints of a soapy albuminous fluid, and several hydatids. The *observations* of Dr. M'Loghlin do not contain an original, useful, or interesting idea. We were sorry to see so many pages (45) of this valuable volume occupied by the narration of this case; which is done with the most tedious prolixity.

*Observations on the Epidemic Fevers of Dublin, founded on a Report of the Hardwicke Fever Hospital, during the Years* 1813, 1814, *and* 1815; by EDWARD PERCIVAL, M.B. Cantab. and Dublin, M.R. I.A. &c.

The length of the period embraced by this report, and the extensive field opened to the observation of Dr. Percival, made us anticipate much of the pleasure we have derived from the perusal of this paper. It is only by the contemplation of an epidemic under all the changes of season, and distinguishing the features of generic resemblance, from the varieties which present themselves influenced by the difference of those seasons, (a mode of observation so much insisted on by Hippocrates and Sydenham,) that those fundamental principles of pathology can be ascertained, which are applicable, under certain modifications, to every variety of the epidemic disease. Such are the views from which the following observations were deduced.

Our readers will bear in mind, that this report embraces a period, at which the people of Ireland were suffering the greatest distress from poverty and famine; large tracts of land lay uncultivated from the inclemency of the weather of some preceding years, and the change which had taken place in the commercial transactions of the country having rendered labour unprofitable. The consequence of this was, that great numbers of idle persons flocked into the capital, to seek there for sustenance they could not elsewhere obtain. An epidemic fever, appearing under such circumstances, may be supposed to have committed extensive ravages, and assumed a character which would justify the alarm it excited in the minds of the inhabitants.

"The *season* of greatest pressure for admission to the Hardwicke Hospital, (says Dr. P.) was usually from the commencement of spring until midsummer. Whatever be the causes which generate or diffuse epidemic diseases, it appeared, that among the adult poor in the House of Industry, and still more remarkably among the children, crowded as they all were, (from the distresses of the times,) in very inadequate dormitories, fever spread with peculiar facility on the dawn of mild weather, after the severity of winter. I could even trace this fact in the strongly marked vicissitudes of spring, which belong to this climate. A single week of soft or sultry weather, after a prevalence of sharp winds and cloudy skies, was followed by manifestations of fever, requiring prompt vigilance to control its progress.

"The subjects of the *vernal epidemic* were chiefly the young and the robust. If persons past the middle age were involved in it, they were generally such as had impaired constitutions, or diseased pulmonary organs. Yet many cases of petechial and typhoid fever occurred without distinction of age; parents and children being transferred to the hospital at the same time, labouring under similar diseases."

The epidemic of this season was more generally inflammatory, or, as the French express it by a compound Greek word *angeiotenique*, than that of any other period. Catarrhal and peripneumonic symptoms prevailed in the early stages; yet the disease seldom began with acute pains in the chest, but rather oppressed respiration from inflammation of the mucous membrane of the bronchiæ. The greater number of cases being of a mild kind, the average term of the fever was short; it seldom exceeded nine days. The mortality was small, alighting almost exclusively on phthisical subjects or depraved and worn out habits. The typhus of modern writers was prevalent in the spring; and, though it declined as the season advanced, yet specimens of the worst kind continued frequently to appear as intercurrents. Children frequently exhibited minute dusky petechiæ, about the trunk of the body particularly. These quickly disappeared under the cool regimen and purgative discipline of the hospital. The complication of peripneumony, with the extreme symptoms of typhoid debility, was more frequent in the spring than any other season.

The *summer* soltice produced no sudden change in arresting the epidemic, or converting its character. Catarrhal symptoms continued, but pneumonic distress gradually declined, as the warm weather grew confirmed. Pain and heat of the stomach, with great tenderness of the epigastrium, began to prevail. Retching or vomiting of bile and mucus sometimes occasioned much distress to the patient, who appeared to labour under peculiar feelings of irritation and despondency. The right hypochondriac region, and the epigastric, so far as the umbilicus, became soon engaged with soreness and oppression; the bowels were constipated, the skin dry and hot; yet the pulse was feebler, and the angiotenic character less observable than in the preceding epidemic.

"These symptoms were marked most clearly in patients of middle age, among whom this fever prevailed chiefly. Its duration depended much on the period at which the patient was admitted to the hospital; yet the term of acute fever did not often exceed nine days, unless when it was complicated with tympany, singultus, insomnium, and other alarming symptoms."

The vague term, bilious fever, commonly applied to this species, is justly reprobated by the French writers. M. Pinel proposes to substitute the appellation "meningo-gastrique." "Every thing," (he says) "seems to indicate that the principal seat of the diseases of this kind is in the alimentary canal, particularly the stomach and duodenum, rather than the secretory organs of the bile and pancreatic juice." (*Nosog. Philos.* tome i. p. 90). Dissection generally shews the consequences of inflammation of the mucous membrane of the alimentary canal, and dropsy not unusually follows the more protracted cases, from a conversion of the inflammation to the serous membranes of the abdomen.

"The *autumnal* epidemic (which generally extended itself into the winter) partook of the character of the antecedent fever, in derangement of the mucous membrane and hepatic viscus. But the seat of peculiar congestion in the autumnal fever, was the inner surface of the intestines, and sometimes the mesenteric organs. The type of this epidemic was more irregular than any other; its invasion more obscure; its progresss and duration less defined. The subjects of the disease were often broken and declining constitutions, in which the digestive organs had long been impaired; and, under these circumstances, if the patient escaped the first violence of febrile excitement, he incurred no less hazard, from imperfect crisis, relapses, hectic fever, and marasmus. Under the pressure of typhoid symptoms, also, the fever proved dangerous; but the young and the robust usually experienced the disease in a mild form. Women, if they were not dram-drinkers, bore it more favourably than men; and amongst these latter, the danger was greater in proportion to their age and visceral infirmities. Though agues prevail in some parts of the neighbourhood of Dublin, yet the patients are rarely sent to the hospitals of the city; and neither my notes, nor my recollection, furnish any examples of the autumnal synochus degenerating into the true intermittent fever.

"The worst forms of typhous fever prevailed at an advanced period of the *winter*. Livid blotches, a dry tongue, dark and tenacious mucus on the gums and lips, muttering delirium, singultus and lethargy were frequent symptoms. Peripneumonic distress attended, at least the commencement of most of these cases. The hepatic viscus was also frequently engaged. But the peculiar seat of sanguineous congestion appeared to be the brain and its investing membranes.

All ages, except infancy, were liable to this fever; the duration of which, under the circumstances above described, seldom fell short of fourteen days, and often exceeded seventeen. It is needless to add, that it proved more frequently fatal than any other form of epidemic or contagious fever.—Danger was to be apprehended under all circumstances of bodily constitution; yet the fatal cases which I had an opportunity of exploring by dissection, generally exhibited, besides cerebral congestion, extensive visceral disease.

"Of the fatal cases which occurred to my observation in the Hardwicke Hospital, the greater number exhibited, on dissection, evidences of antecedent visceral disease kindled into new actions, or the suppurative process, by the violence of febrile circulation. The lungs, the liver, the mesentery, and mucous surface of the primæ viæ, were the parts chiefly implicated in this destruction; and the character of the reigning epidemic, commonly (but by no means invariably) determined the organ thus engaged. Hence the infirm, whether from long hardship, intemperance, or morbid constitution, fell a sacrifice to epidemics, which the young and robust weathered through, with little injury or danger."

After some remarks on the relative proportion of males and females, on the ages of the patients, and the season at which the greatest numbers were admitted, which we do not enter into the consideration of, as depending in a certain degree on local causes, Dr. P. adverts to the question respecting the contagious nature of fever. He considers that epidemic fevers, in large towns, do, under certain circumstances, become contagious. "Yet," he observes, "on the other hand, sporadic cases do unquestionably appear, under such insulated circumstances, as to preclude all proof or presumption of their contagious origin." A question then arises, whether these apparently spontaneous cases differ in their symptoms or nosological character from other concurrent cases which are referrible to contagion? Many endeavours have of late been made to disembarrass the science of medicine of these questions, by various artificial and hypothetical arrangements; but Dr. P. says, no recent authorities have cancelled his assent to the opinions of Willis, Huxham, Grant, and Pringle. He enters at some length into the consideration of the opinions of some modern writers on this subject; but, from their having already been brought before our readers, and fully discussed, we pass them over in this place. The author then observes, that, having enquired into the origin of most cases of fever that were admitted into the Hardwicke Hospital, during several years, he found they pointed less frequently and precisely to a contagious source than he should have anticipated. And he continues to remark, that "no peculiarity of symptoms or sequel distinguished the fevers which could (apparently) be traced to contagion, from those which could not be so referred."

Fever frequently sprang up and spread among the inhabitants of the Bedford Asylum, (a building for the reception of poor children,) during one or two years, when it was much crowded, and the dormitories, in particular, quite inadequate in size for the number contained in them; but, since the numbers have been thinned, and the dormitories more effectually ventilated, fever has almost wholly disappeared.

The division of epidemic fevers into genera is considered improper; the resemblance, or convergency, of *typhus mitior* and *synochus*, are at least as palpable as the same relations between *typhus mitior* and *typhus gravior*, (these terms are necessarily used for want of better being generally understood.) Each species commences with some inflammatory congestion, and each will terminate in the same way. They occur indiscriminately in the same season, district, and different members of the same family; and the gradations from the mildest to the severest type depend *principally* on early or late removal from crowded and ill-ventilated dwellings.

The following observations of Dr. P. will more fully develop the above sentiments.

"The leading features of epidemic and contagious fevers are rapid prostration of strength, with sanguineous determination to the head, or other principal organs, attended with frequent pulse, and suspended or disordered secretions. The strong analogy prevailing amongst all fevers of this description, seems to indicate a community of generic character. They differ from fevers arising from simple local inflammation, in many important particulars; but in none more remarkably than in the sudden failure of mental and voluntary power, and in their uniform tendency to perform a certain cycle of morbid changes, in definite periods."

The worst forms of fever seldom appeared in young subjects. In middle-aged persons, the most formidable cases were those which had been neglected during their early stages, or which, occurring in depraved habits, involved severe organic disease. Fevers which had been preceded by great bodily fatigue or mental anxiety, were uniformly hazardous, observing the *atactic* character of the French nosographers. This "*fièvre typhode ataxique*" seldom appeared among the lowest order of labourers; it rather shewed itself among struggling or decayed tradesmen and artizans.

The worst symptoms of fever, observes Dr. Percival, are pervigilium, tympany, singultus, coma; the most favorable, in all cases, are sleep, a moist tongue, and solvent bowels. The state of the pulse he considers a much less uniform or satisfactory criterion than it has usually been esteemed. The appearance of petechiæ is still less subservient to prognosis. The countenance and posture of the patient, his manner of respiration, and the appearance of his tongue, give various and authentic information to the experienced practitioner. The tongue may be moist, and thinly coated with white mucus, as in the febrile phlegmasiæ; in which case the temperature of the body is usually high, and some viscus engaged in active inflammation. When it is thickly covered with yellow mucus, the hepatic viscus is in a state of congestion. A brown dry streak in the middle of the tongue indicates intestinal torpor and defective secretion throughout the canal. A dark, dry, and shrunk tongue, with difficulty protruded beyond the teeth, indicates a deeply vitiated condition of the secretory organs, with extreme prostration of forces, both animal and vital. The tongue sometimes has a high florid hue; this obtains chiefly when the fever has become hectical from suppurating surfaces, or when dysenteric disease has formed.

The duration of the severer forms of fever was seldom less than fourteen days. "The crisis was commonly marked with a distinctness." The most favourable was attended with a deep sleep, protracted sometimes for the space of forty or fifty hours, with brief intervals. The critical period was often a scene of severe struggle. An obscure rigor would set in on the eve of the fourteenth day, or later, delirium and jactitation would encrease, the extremities become cold, respiration hurried and oppressed; the countenance pale and anxious; and the pulse, by its frequency, smallness, and irregularity, scarcely numerable: the patient would moan from pains referred to the back and limbs. This struggle continued for some hours, and then subsided into relief or the gradual extinction of life. Relapses were rare in the hospital; they were sometimes threatened by errors and excess in diet; and were generally prevented by an emetic and purgative.

The most common period of mortality was between the eleventh and seventeenth days. Examination of bodies after death generally exhibited marks of inflammation or congestion in the brain, lungs, liver, peritoneum, or the mucous texture of the intestinal canal. The liver was frequently diseased, especially in the summer and autumnal fevers. The class of persons who were the subject of examination must here be considered, many of them being long habitual drunkards. A vein of predominance was observable in the morbid appearances in each season. Pulmonic disorganization was chiefly detected in the spring; gastric derangement in the summer; hepatic and enteric disorder in the autumn; and cerebral congestion in the winter.

We pass over some remarks on the various theories of fever which have at different times been advanced, and some particular observations on the pulse, petechial eruptions, &c. to the consideration of the *curative treatment.* The first thing to be insisted on is a plentiful supply of cool and fresh air. It is one of the most powerful auxiliaries in tempering morbid heat, allaying irritation, banishing petechiæ, and promoting sleep and moderate diaphoresis. The next point to be regarded is suitable evacuation, and, primarily, that of blood-letting; but we may pass over the arguments on the propriety and necessity of this practice; only observing, that the opinions of Dr. P. on this subject concur with those of Jackson and Armstrong.

In the typhous epidemic of summer, attended with morbid secretion of the abdominal viscera, a high temperature of the body, and little disturbance of the respiratory organs, cold affusion, followed by purgatives, superseded the use of the lancet in the greater number of cases. The afternoon, or evening, Dr. P. observes, appears to be the most favourable time for bleeding patients in fever, as it is usually the period of diurnal exacerbation. This rule, however, is not intended to restrain the use of it at other times, when it is manifestly required. It may be a useful caution to observe, that the remissions of the morning do not always furnish safe indications for practice in this respect.

Cold affusion and ablution was extensively used. The younger patients, who were free from visceral disease, and who were admitted within the third or fourth day, were treated with cold affusion, which, aided by a few doses of purgative medicine, effected a rapid and almost invariable cure. In full-grown subjects, from the difficulties in many instances attending the process of removing them from their beds, against their will, constraining them to sit in tubs, &c. it was rendered necessary to substitute cold ablution for affusion.

In the catarrhal fever, and in those cases of peripneumonic fever which required the free use of the lancet, affusion was never employed, but the sponge was sometimes used with benefit. The common kind of vernal fevers, with pulmonic oppression without inflammation, were relieved or cut short, without any hazard, by cold affusion.

Active purgative medicines were freely exhibited; they were particularly employed when the tongue was loaded with yellowish mucus, or when it exhibited a dark and shrunk appearance; so long as the stools were pitchy, of a black or greenish hue, and either preternaturally fœtid, or unusually inodorous, no remission of suitable cathartics was allowed.

Vomits, when given on the first or second day of fever, and followed by cathartics, sometimes cut short the disease, and in most cases mitigated the symptoms. Now and then, during convalescence, a slight accession of fever, threatening a relapse, was obviated by an emetic; but they did not form any part of the stated or ordinary practice.

Diaphoretics, in the acute stages of fever, were not productive of benefit; after a full trial of antimonials, they appeared serviceable only when they excited vomiting or purging. But in those varieties of fever protracted by imperfect crises, when the tongue remained coated, and the skin dry and hot, antimonial powder was given with advantage. Cold water, as a drink, was left to the discretion of the patient.

Of the use of mineral acids in typhous fever, Dr. P. observes, he has little experience, from the incompatibility of administering them freely in conjunction with calomel, on which much of his reliance was placed in almost all the worst forms of typhus.

Cinchona was seldom given, except during convalescence. Whenever visceral congestion prevailed, when the tongue was loaded with mucus, or dry and shrunk, it was decidedly injurious: as an antipetechial remedy, it was useless. It did not check the tendency to gangrene of the nose or extremities of the feet, or contribute to remove livid blotches.

Of all cordials, wine was the most salutary; a weak grog (made with whiskey) was frequently substituted for it, and appeared to answer the purposes intended.

Of the use of opium, as a cordial or general remedy in typhous fever, Dr. P. says he has little experience, and such as gives him no encouragement to extend it. When the belly is costive, and the temperature high, its effects, like those of wine, are certainly pernicious; but, when fever has been preceded by, or is accompanied with, much mental solicitude, and the patient is continually tossing about in the bed, when there are no signs of local congestion, and the skin is not very hot and dry, we have found opium highly beneficial; it will then procure rest, gentle diaphoresis, and wonderfully quiet and recruit the system.

Blisters were found to be seldom admissible until due evacuations had been premised. Dr. P. generally directed them to be applied to the back of the neck in congestion in the head, that they might not interfere with cold ablution. They were highly useful in the latter stages of pulmonary congestion. Low delirium, stupor, and pervigilium, are indications for their use; in which he was seldom disappointed of at least temporary benefit. A blister applied on the eve of a critical day, he says, often appeared to determine a favourable change, under very unpromising circumstances.

We have passed over many valuable practical remarks in this paper, both on the symptoms and treatment of fever; from their concurring with those we have so lately brought before our readers in our reviews of the judicious and erudite works of Armstrong and Jackson.

(*To be concluded in our next.*)

---

*Edinburgh Medical and Surgical Journal*, No. LV. for July, 1818.

Art. I.—*Observations on Chronic Inflammation of the Brain and its Membranes.* By John Abercrombie, M.D. Fellow of the Royal College of Surgeons of Edinburgh.

This, as our readers will anticipate from the well-known abilities of the author, is a valuable paper, containing many useful practical remarks. We, however, consider that an attempt at nosological arrangement of the diseases of the brain has led Dr. Abercrombie into some errors.—He says,

"Diseases of the brain may be divided into three classes, the inflammatory, the apoplectic, and the organic. Active inflammation of the brain is in this country so uncommon, that some have doubted whether it really exists as an idiopathic disease. For this reason, I confine my observations to chronic inflammation. I include under this term all those affections of the brain, which, beginning with symptoms of an inflammatory nature, terminate either by suppuration or effusion; and I do not comprehend serous apoplexy, which, beginning with apoplectic symptoms, belongs to another branch of the subject. Those affections which I include under chronic inflammation appear under various degrees of activity. Some of them are evidently examples of the pure scrofulous inflammation, while others approach to the characters of acute phrenitis, and on this account there may be some objection to the term. But, as they pass into one another by almost insensible gradations, and are intimately allied in their symptoms and their terminations, and, as none of them exhibit all the characters laid down by systematic writers, as those of Phrenitis, it appears to me, that it will simplify the subject, if we consider them all under the general term chronic inflammation."

We by no means concur with the opinion, that active idiopathic inflammation of the brain is such an uncommon disease in this country that its existence may be doubted; and, although Dr. Abercrombie literally expresses a different opinion, do not the facts he relates show that his observations should lead him to agree with us? He states that chronic inflammation of the brain is a disease of frequent occurrence, and that "chronic inflammation appears under various degrees of activity; some cases approach to the character of acute phrenitis."

The word *approach* may, perhaps, in this instance, be used with the same latitude, as those in which the term *general* is so often employed, when writers have not determinate ideas on their subject; but we can understand nothing from the above paragraph, but that the diseases it would describe are the same, only differing in degree. Neither do we concur with the author in his opinion, that, because a disease does not exhibit *all* the characters by which it is described by a compiler of a system, that it is to be considered as a distinct disease, when the general symptoms are the same. This is a point of practical importance; the adoption of the distinction of Dr. Abercrombie, in diseases in which it is evident a difference cannot be demonstrated, would induce many practitioners to delay employing those measures, on the early or late adoption of which, the life of the patient will so often depend. We shall make some remarks on the term *scrofulous* inflammation of the brain, when we consider another part of this paper, in which it is used in a still more objectionable manner. Dr. Abercrombie divides diseases of the brain into three classes; the inflammatory, the organic, and the *apoplectic*. We are at a loss to imagine how the latter term can be made to designate any distinct class of diseases. Apoplexy is a mere consequence of a diseased state or action, and may ensue from the diseases which may be comprised in either of the other classes.

Chronic inflammation of the brain, he thinks, may be referred to four classes.

The *first* form of the disease most commonly affects children, but may also appear in adults. It is usually preceded for a day or two by langour and peevishness; the accession of fever is ushered in by a severe shivering: the patient is oppressed and unwilling to be disturbed; complains of severe pain in the head and impatience of light. In some cases there is vomiting. The pupil is usually contracted; the tongue white, but moist, sometimes clean; sleep is disturbed by dreams. After a few days, delirium begins to appear; a tendency to sleep follows, which soon passes into coma. While these changes are going on, the pulse, which was at first frequent, usually falls to the natural standard or below it; the eye becomes dull, vision obscure or double, and the pupil is dilated. The pulse, having continued slow for a day or two, begins to rise again, sometimes to two hundred in a minute: the patient is now in a state of perfect coma, sometimes accompanied by paralysis of the limbs, at others by convulsions: after he has continued in this state for a few days, death ensues. The duration of the disease is from one to three weeks. At some period of the disease, there is generally a remarkable remission of all the symptoms, which gives sanguine but deceptive hopes of recovery; it usually occurs as the pulse is falling in frequency, or when it is beginning to rise after the slowness, and is the prelude to coma.

The *second* form Dr. A. has most commonly observed in young persons towards the age of puberty: it begins like a slight feverish disorder, and for a considerable time excites no alarm. There is slight head-ache, disturbed sleep, general uneasiness of the limbs, and impaired appetite; the tongue is foul, and the pulse from 90 to 100: remissions and re-accessions occur for several days. After the sixth or seventh day, the head-ache becomes more severe, and is more constant than corresponds with the general symptoms of fever: a sense of oppression is also then observed; sometimes the tongue becomes clean; and, towards the twelfth or fourteenth day, the pulse falls to the natural standard, while the head-ache is increased, with a tendency to stupor; convulsions and double-vision frequently appear: the pulse then begins to rise again, and there is frequently a deceitful interval of amendment; but the patient soon relapses again into perfect coma, and dies in three or four days. The duration of the disease is from two to six weeks.

The *third* form of the disease usually affects adults. It begins with violent head-ache without fever; the patient lies in bed, oppressed, and unwilling to be disturbed, or tossing about from the violence of the pain; the pulse is about the natural standard, sometimes below it; the face sometimes flushed, at others pale; the pupil is usually contracted, and there is impatience of light. Sometimes delirium appears at an early period, varying in degree until in six or seven days it degenerates into coma; the pulse having continued through the whole course of the disease from 70 to 80. In other cases, the pulse is at first about the natural standard, afterwards falls to 60 or 50, and at last rises to 120 or 130. There is in every case more or less delirium, but it is often slight and transient; sometimes the patient lies in a dosing state, with incoherent muttering, but can be roused to talk sensibly. This condition when not accompanied by fever, is always characteristic of a dangerous affection of the head. There is frequently a forgetfulness and confusion of thought, of which the patient is himself sensible; the speech is sometimes affected, and this may either be from a difficulty in articulation, or a hesitation from the patient not being able to recollect the word he meant to make use of. There is generally towards the end more or less coma, but sometimes the disease is fatal without the intervention of coma, the patient being able to answer questions distinctly until a short time before his death.

The *fourth* is a very frequent form of the disease. The first symptom that excites alarm is a sudden and violent attack of convulsion; sometimes this is preceded by vomiting, at others by head-ach for several days. In some cases, the convulsion is followed by coma, which in a few days is fatal; in other cases, the convulsion recurs after short intervals, the patient in the intermediate time complaining of head-ach. The convulsion is occasionally confined to one side of the body, or to one extremity, and is usually followed by paralysis of the part.

Dr. Abercrombie makes some judicious remarks on the nature and cause of those symptoms, and shows the importance of watching them with caution, that we may not be deceived by false appearances of amendment. Chronic inflammation of the brain, he observes, is not always an idiopathic disease; it often takes place in the course of other diseases, the most common of which are continued fever, scarlatina, measles, pneumonia, phthisis, and diseases of the kidnies. It will be useful, therefore, to keep in view those symptoms which, in the course of any disease, indicate a tendency to this dangerous affection of the brain.

The terminations of chronic inflammation of the brain are referred to the following heads:

1. The disease may be fatal in the inflammatory stage.

2. By serous effusion, either into the ventricles or on the surface of the brain.

3. By suppuration.

4. By a peculiar destruction or disorganization of the central parts of the brain; as the fornix, septum lucidum, and the white medullary matter which lines the ventricles. The parts appear as if broken down into a white soft pulpy mass, retaining their natural colour, but losing their figure and consistence.

5. Deposition of coagulable lymph.

6. Thickening of the membranes, contraction of the sinuses, caries of the bones, and other affections of the external parts.

Many examples are related of each of those modes of termination.

Under the head of *Causes and Treatment of Chronic Inflammation of the Brain*, the author says, "In its least active form, the disease is an example of the pure scrofulous inflammation." Whether scrofulous inflammation be merely *chronic* inflammation of the most indolent kind, or *specific*, may admit of a doubt; but, from some remarks which ensue, it would appear that Dr. A. considers it as *specific*; if so, it is very rude pathology to apply the term to the disease in question, and such as cannot be supported by facts or theoretical induction: the author continues with stating, that—

"Scrofulous inflammation is often excited by very slight causes, and often appears without any cause that we can trace. On the surface of the body, we see it excited by very slight injuries, which in a healthy constitution would produce no bad effect. It frequently follows altered determinations of blood: thus I have seen suppression of the menses in a young woman of a *scrofulous habit*, followed immediately by extensive abscess in the mamma. *Scrofulous* or *chronic* inflammation also appears in combination with a variety of febrile complaints, as if the mere febrile state brought it into action. In this manner we meet with it affecting the lungs, the bowels, and glandular parts, in continued fever, and in scarlatina. These observations apply to chronic inflammation of the brain."

We must confess we cannot understand what is the author's meaning in the above passage, or form a clear idea of what his opinions are respecting scrofulous inflammation. In one sentence we are led to suppose that he considers it a species of inflammation *sui generis*; and, in another, he uses *scrofulous* and *chronic* as synonymous terms. It cannot be allowable in medical, any more than in general philosophical, reasoning, to employ terms in a new sense without having previously explained their precise meaning, or argue on propositions which are not supported by facts.

From observing, in a variety of instances, that serous effusion in the brain had taken place to a great extent without inducing symptoms or pressure, or those which are usually attributed to it, Dr. A. is inclined to think that those symptoms do not arise from the presence of the effused fluid, but from the state of brain which induced it. The following are the general conclusions deduced:

"1. That in cases of hydrocephalus, the coma and other symptoms are not to be considered as the direct effect of the effusion, but of that morbid condition of the brain of which the effusion is the consequence.

"2. That we have no certain mark which we can rely upon as indicating the presence of effusion in the brain. Slowness of the pulse, followed by frequency, coma, squinting, double vision, dilated pupil, and paralytic symptoms, we have seen, may exist without any effusion.

"3. That these symptoms may exist in connexion with a state of the brain which is active or simply inflammatory, while the disease is the subject of active treatment, and while by such treatment, adopted with decision and promptitude, we have the prospect of arresting its progress in a considerable proportion of cases. The ground of prognosis in particular cases is obvious. The more they approach to the character of acute phrenitis, the prospect of cutting them short will be greater; and the more they partake of the pure scrofulous inflammation, it will be the less. In all of them, the period for active practice is short, the irremediable mischief being probably done at an early period of the disease."

II.—*Additional Observations on the Cure of Syphilis without Mercury.* By John Hennen, Esq. Deputy-Inspector of Hospitals for North Britain.

The observations contained in this paper add to the importance of those before made by the author on this interesting subject. The applications to the primary sores usually employed have been cataplasms, astringents, stimulants, and in some cases the solution of arsenic, according to casual circumstances. In all cases, rest in the horizontal posture is an important part of the cure. Of those ulcers having the Hunterian character of chancre, the time usually required for the cure was from ten to thirty days; those not having the Hunterian character of chancre required an equal, and not unfrequently a greater, length of time to be induced to heal. Secondary symptoms occur more frequently, and appear at an earlier and more determinate period than where mercury has been used; but they, in many cases, have gone off as soon, never, as has been supposed, proceeding from bad to worse, or from one succession of parts to another, with unabated violence; on the contrary, they by no means exhibit the same violent and unrelenting symptoms observed in many instances where mercury has been used: the eruptions have not run into ulceration, nor have the bones of the nose, or of other parts, been in any instance affected with caries.

Whatever may be the result of this investigation, we trust that it is conducted in a manner which will not permit criminality to be attached to its directors, should the opinions attempted to be established prove erroneous.

VIII.—*On the Connection between the Symptoms of the Fever which prevails at present in Glasgow, and a Morbid Affection of the Brain and Spinal Marrow.* By JAMES SYM, Surgeon in Kilmarnock, and lately House-Surgeon to the Glasgow Royal Infirmary.

It is with no small degree of satisfaction we observe that the spinal marrow is beginning to be generally attended to in *post mortem* examinations. The opinions and observations of Dr. Franck, (adopted and extended by Dr. Sanders of Edinburgh and Dr. Reid of Dublin,) have much contributed to direct the attention of pathologists to this part of the nervous system; and, from the facts already discovered, we may thence anticipate much important addition to our pathological knowledge.

The unsatisfactory information derived from examination of the brain, in persons who have died of continued fever, induced Mr. Sym to extend his enquiries to the spinal marrow, and he, in almost every instance, found the consequences of inflammation, and particularly effusion of serum, to a considerable extent. After noticing the symptoms in continued fever, which appear to depend on a diseased state of the brain, the author observes,

"Other symptoms, however, which seem to depend on a deranged action of the nerves arising from the spinal marrow, occur at the same time, and modify the features of the disease. There is a sudden loss of power in all the muscles; the patient has a sensation of uneasiness over his whole body, which he is unable to describe; he is fatigued, and muscular exertion becomes as irksome to his feelings, as their natural stimuli are to the organs of sense. The pain shoots from his back along the lower extremities, and diffuses itself slightly over his whole body, becoming less acute as it recedes from the spinal marrow, and changing into a general soreness or sense of bruising. There is a simultaneous derangement in the functions of all the viscera, however remote from each other in their situations, and distinct in their offices. The stomach cannot retain its food, or at least cannot digest it; the bowels become costive, and the stools morbid; the limbs are affected with convulsive twitches; the bladder is paralysed; all the sphincters are relaxed; the cellular substance of the hips dies; and the feet and legs become cold and fall into gangrene, in the same manner as when the principal nerves are compressed by a ligature. The symptoms differ so much from debility from a lingering disease, that I think they must depend, in a great measure, on the pressure of that serum we so uniformly meet with in our dissections."

The following morbid appearances are considered by Mr. Sym as particularly connected with typhous fever.

"There is a sudden loss of power in all the muscles; the patient has a sensation of uneasiness over his whole body, which he is unable to describe; he is fatigued; and muscular exertions become as irksome to his feelings, as their natural stimuli are to his organs of sense. The pain shoots from his back along his lower extremities, and diffuses itself slightly over his whole body, becoming less acute as it recedes from the spinal marrow, and changing into a general soreness or sense of bruising. There is a simultaneous derangement in the functions of all the viscera, however remote from each other in their situations, and distinct in their offices. The stomach cannot retain its food, or at least cannot digest it. The bowels become costive; the stools morbid; the urine high-coloured; the mouth parched with thirst; the skin hot; and the respiration and motion of the heart hurried to twice their usual frequency. When several organs, which do not communicate directly with each other, have their functions thus impaired at the same instant, it is difficult to avoid concluding, that the common source of those nerves, by which they are all supplied with energy, has ceased to perform its duty in a healthy manner."

IV.—*Account of a singular Rheumatic Affection, of an Intermittent Type;* by NATHANIEL RUMSEY, Member of the Royal College of Surgeons in London.

THE author relates several cases which appeared to be rheumatic affections of the abdominal muscles, accompanied by a disordered state of the bowels, sickness, flatulence, &c. that were cured by the use of cinchona.

V.—*Case in which the Nasal Operation has been recently performed;* by W. HUTCHINSON, late Surgeon to the Royal Naval Hospital at Deal, Surgeon to his Royal Highness the Duke of Clarence, and to the Westminster General Dispensary.

THE subject of the operation is a married woman, aged about thirty-two, who had lost every part of her nose, including even the nasal bones, about eight years ago,—as well as could be learned, from gangrene succeeding to an attack of erysipelas.

The operation was performed without its being necessary to tie one blood-vessel. A needle and ligature were passed through the upper lip and new septum, and two on each side of the nose, to retain the newly cut surfaces in contact. On the second day, previous to the dressings being removed, a slight hæmorrhage occurred from the posterior or internal surface of the new nose, the blood escaping by the mouth. The stitches were cut away on the sixth day; and on the eleventh from the operation, there was external hæmorrhage to the amount of about thirty ounces from the right angular artery, but which stopped spontaneously: the blood issued from the part whence the stitch had been removed. Every part of the nose externally is now cicatrized; the wound on the forehead diminished to the size of a shilling; and the nostrils are kept pervious by the introduction of a sponge tent.

VI.—*On the Mode of performing Arteriotomy;* by CHRISTOPHER KANE, Member of the Royal College of Surgeons, London.

FROM the style in which this paper commences, we expected to find something in it new and important; but it is "*fumum ex fulgore.*" There is nothing new in Mr. Kane's mode of opening the temporal artery; nor are the entire division of the vessel, after having abstracted a sufficient quantity of blood, and the application of a ligature instead of compression, unusual measures.

VII.—*Case of Hemicrania, cured by the Application of Leeches;* by D. ROBINSON, Member of the Royal College of Surgeons in London.

THIS was a case of pain affecting the parts throughout the whole course of the *pes anserinus*, apparently connected with suppuration in the *meatus auditorius;* that was relieved by the use of leeches and blisters.

VIII.—*A peculiar Case of enlarged Ovarium, associated with Mental Derangement;* by G. F. EDWARDS, Member of the Royal College of Surgeons in London.

A case possessing no peculiar interest, nor from which any practical information can be derived.

*An Essay to improve the Method of performing certain Surgical Operations, and provide Instruments for the Purpose; to which are added some Forms of Bandages, chiefly for Fractures.* By William Jardine, Surgeon of the Royal Navy. 8vo. pp. 180. Nivison, Edin. Highley, London.

These seem to be the observations of an experienced practitioner, possessing considerable mechanical talent, which he has applied to the improvement of surgical instruments. The deficiency in instruments being, as he states, "one reason why some important operations are performed in a very different way to what they would be, were surgeons supplied with instruments fitter for their purpose than they are."

We are not advocates for increasing the number or complication of instruments, being persuaded that the success of an operation depends more on the operator than the instrument. In the course of our experience, we have seen many ingenious contrivances used by none but the inventors, and by them only while the charm of novelty lasted: indeed, almost every surgeon has some peculiarity of shape or make to suggest, while the true master of his art is less scrupulous on this head; and we remember Mr. Hunter to have said, that, with a scalpel and a probe, he could travel over the whole body.

In this Essay, Mr. Jardine has given many useful suggestions, to which the young practitioner will do well to attend, particularly those who are called early into public service.

Of the real merit of mechanical improvements, it is impossible to judge, without practical experience, of their application; and, as we are deficient in this knowledge, we can only presume that they have, in the hands of the inventor, answered all the useful purposes he suggests.

As it would not be possible to convey to our readers clear ideas of the construction of those instruments without graphical illustrations of them, we shall confine our notice to one or two, of which the advantages are most evident, and may be most readily explained by mere verbal description.

The first is an improvement in the trephine.

"This instrument is shaped in the form of a common trephine, with two rims upon its crown or barrel, the one above the other. The upper rim is about three-quarters of an inch broad, and one-sixteenth thick, and is screwed on; the under is about half an inch broad, and one-sixteenth thick, and moves easily a little up and down upon the crown. They are separated by a spiral spring round the crown betwixt them, which above is fixed to the upper rim, and below to the lower one; the under rim is made to rise or approach the other about the eighth of an inch, by pressing it up against the spring; but, if required higher, or beyond the contraction of the spring, the upper rim is screwed up the height requisite, and the lower one, from its connection with it, also rises.

"This instrument is adjusted by the upper rim, which is screwed up or down to raise or depress the lower one; so that the lower one, at first, may be made to stop close to the teeth of the saw, or thereabouts, allowing them (the teeth) to project no further beyond it than what is just necessary to keep the saw in its place while working.

"The expansion of the spring keeping down the under rim, prevents the saw from being suddenly forced in upon the brain, yet yielding sufficiently and gradually, permits it to rise proportionately to the progress of its teeth into the bone, and in such a way that there is no risk of their being inadvertently forced in too far. The teeth having now penetrated the bone about the sixteenth part of an inch, or proportionably to the expansion of the spring, the upper rim is screwed up about the sixteenth of an inch higher to permit the under one to rise also; so that the saw may penetrate still farther, which is to be wrought again as before, and so on until the bone is cut through."

This instrument may certainly possess advantages over the trephine generally used, in cases in which the internal table of the cranium is more extensively fractured and depressed than the outer; in the hands of an inexpert operator; and in performing the operation at sea by those unaccustomed to do it under such circumstances.

The instrument for extracting foreign bodies from the œsophagus is apparently well adapted for the purpose.

"It is made of five or six threads of catgut, nearly three inches long, twisted round the end of a wire that passes through a flexible pipe, and protrudes about two inches and a half beyond its extremity; one end of the twist is fixed to the end of the pipe, and the other to the end of the wire. In its extended form, it is to be introduced so far into the throat that all the catgut may be supposed to have got beyond the bone or thing to be extracted. The ring at the end of the handle, and outside of the mouth, is then to be drawn out about an inch to spread the catgut, and, in withdrawing the instrument, the bone will be extracted with it."

---

*An Essay on the Symptoms, Causes, and Treatment, of Inversio Uteri; with a History of the successful Extirpation of that Organ, during the Chronic Stage of the Disease.* By W. Newnham, Esq. 8vo. pp. 152. Cox, London.

We have too much cause to regret the little practical knowledge we possess respecting some of the most distressing maladies to which the human body is liable, because they may not be of such frequent occurrence as to afford to any individual the means of deducing useful information from his own opportunities for observation. A few cases, widely dispersed, or perhaps collected by compilers, often without including the circumstances that would render them useful, being all that literary records afford. It is only from extensive series of facts that general conclusions can be drawn; when these are wanting, each individual must act solely from the dictates of his own judgment; this necessarily gives rise to various modes of proceeding, which, with their consequences, embarrass rather than render assistance to the young practitioner. Such is the case with respect to the subject of this work. Every endeavour, therefore, to add to our knowledge, and to remove unjust alarm respecting a mode of treatment, which is often the only one that will render life tolerable to the patient, must receive the approbation of the profession.

We shall give the author's intentions in publishing this work, in his own words.

"In presenting this Essay to the medical public, the writer has no interest to serve, but that of suffering humanity; and, had it not been for the purpose of satisfying the most *sceptical* of his brethren, as to the authenticity of the case, he would have infinitely preferred the obscurity of an anonymous publication to the present more obtrusive medium of communicating his facts, opinions, and reasonings. The writer has also to apologise for the number and length of his citations from various authors. Nothing could be more distant from his intentions, than to make a pedantic display of extensive sources of information: but it was of the very essence of his plan, to exhibit not so much his *own conclusions derived from these sources*, as the opinions of others in their naked simplicity; in order to show the *existing state of our knowledge of this disease, to prove the propriety of the operation to be recommended, and to show that such a mutilation might occur without occasioning present or future inconvenience to the patient.*"

Mr. Newnham commences with a concise history of the disease, and the means that should be adopted for its relief. In attempting to reduce the uterus, he advises, if the placenta be adherent, that it should not be removed until this be effected; as the irritation attending the removal of it would probably bring on those bearing-down efforts which would present a material obstacle to its reduction, and increase the hæmorrhage. Besides which, it appears probable, that returning the placenta while it remains attached to the uterus, and its subsequent judicious treatment as a case of simple retained placenta, would have the good effect of inducing that regular and natural contraction on which the safety of the patient depends; should inflammation have taken place, it will, of course, be imprudent to attempt the reduction. Under these circumstances, patients usually die from the irritation and hæmorrhage that ensue. There are some cases, however, where they survive this shock; the uterus slowly diminishes to nearly its natural size, and the hæmorrhage is supplanted by a constant mucous discharge; and the disease assumes that character which forms the principal subject of this Essay.

In treating on chronic inversion of the uterus, the author first considers the palliative measures generally adopted, and observes, that

"From the exhausting discharge which they sustain, such patients require a nutritious diet, and the greatest watchfulness. After all, we shall generally find our efforts unavailing; the woman's comfort is destroyed; and she is going, slowly perhaps, though not less surely, to a premature decease. Under these circumstances, are we not justified, are we not imperiously called upon, to prefer extirpation of the uterus to inevitable destruction? This operation has been repeatedly successful in cases where the uterus has been completely inverted, and it has been removed by an incision or ligature of the vagina; where the uterus has ceased to perform its menstruating functions; and where it has become scirrhous, gangrenous, and very much reduced from its natural size. It remains to be proved, that the same result may be anticipated in cases where the woman has not passed the menstruating age, and the uterus still retains a considerable size."

Mr. Newnham then adverts to the number of cases in which the Cæsarean section has been performed with success, and those also in which portions of the uterus have been removed, in cancer and other diseases of that organ; to show that it may be subjected to considerable violence and irritation, without producing fatal symptoms. He then proceeds to relate the history of a case of successful extirpation of the uterus, the principal facts relative to which are as follows:—The patient, twenty-four years of age, was delivered on the 21st of January of her first child, after a natural labour. The umbilical chord was remarkably short, not exceeding ten inches in length. In about twenty minutes after the expulsion of the child, the placenta was withdrawn from the vagina, where it was felt to lodge. No force was necessary, and the surgeon who attended her, states, that none was used, to bring it away. Upon its removal, however, there was more than an ordinary discharge of blood; and this was observed to gush out from the passage with much force, and as if the source had been much nearer to the outlet than what is common on such occasions. No examination was made at this period. Between this and the following day nothing remarkable occurred, excepting that the patient had not voided any water. The catheter was used daily until the 6th of February. Dr. Davis was consulted a few days previously to the latter period.

"A careful examination soon convinced him of the nature of the disease. The patient was then in a state of considerable febrile irritation. He saw her two or three times; prescribed for the constitutional symptoms, and retired without giving hope or promise of substantial relief to the local affection."

Mr. Newnham first saw the patient in April: she had then a constant discharge from the vagina, of a mucous character, accompanied with frequent hæmorrhage; her countenance was exsanguious; she was daily losing strength; her appetite was declining; and she was subject to frequent and prolonged syncope. The returns of active hæmorrhage were increasing in frequency, and more alarming in degree; and were induced almost by the slightest exertion.

"On examination (says the author) I discovered in the vagina a tumour of considerable size; somewhat of a pyriform shape, larger at its base than at its superior extremity, but not attached by a very narrow neck; surrounded at its apex by the os uteri, between which and the tumour the finger could be readily passed without discovering any immediate connexion; as far as I could ascertain, nearly insensible; and which had never occasioned pain. Hastily concluding this tumour to be polypus uteri, I proposed removing it by the simple application of a ligature. A little subsequent reflection, however, on the case, led me to suspect that it was partial or incomplete inversion of the uterus."

"The history of the case rather countenanced the idea of its being *inversio*, but did not adequately define the nature of the tumour; for it is sufficiently evident that those symptoms might have arisen from the growth of a polypus in the uterus, during the progress of gestation, and which suddenly passed the os uteri after the expulsion of the fœtus."

Mr. Newnham was joined in consultation by Mr. Oke, the result of which was a determination to remove the tumour by a ligature. This was accordingly applied as high as possible upon its neck, taking care to avoid including any part of the os uteri, by carrying the silk considerably within its orifice. It was tightened or slackened according to the indication of the symptoms; and the irritation it produced was allayed by opiates. No symptoms calculated to excite serious alarm occurred. The tumour became detached on the twenty-third day, which proved to be a considerable portion of the uterus; and the patient subsequently regained a good state of health.

The author then proceeds to make some remarks on the uncertainty of the diagnosis of this disease and polypus; he says,

"It is generally remarked, that *inversio uteri* may be distinguished from *polypus* of that organ, by the os uteri not encircling the former tumour in cases of complete inversion, and by the *impossibility of passing the finger around the neck of the tumour, between it and the os uteri, where the inversion has been only partial*, by *the form of the tumour*, *polypus* being *broad at its base*, and attached by a *narrow peduncle*, while the *inverted uterus is broader above than below;* and by the *insensibility* of the tumour in the one case, and by its extreme sensibility in the other; by the *comparative fixity* of the one tumour, and the *extensive sphere of motion* of the other; by the *rough and fungous surface* of inversio, contrasted with the *smooth and polished* circumference of polypus, and by the previous history of the patient's disease."

The author says,

"It is clear that these diagnostics are liable to a great degree of uncertainty, as will appear from the contradictory statements to be presently adduced."

We readily allow, that from each symptom, distinctively, it will not be possible in some cases to distinguish the disease; but we think that, when taken collectively, the cases in which difficulty will exist, are very rare. Adopting the plan, which Mr. Newnham has done, of transcribing passages from numerous writers, theoretical and practical, where distinct symptoms are spoken of as being in certain cases wanting or obscure; it would not be a difficult task to overturn the diagnosis of almost every disease with which we are acquainted. This section of the work, and the following ones on *The Consequences of Inversio Uteri; Prognosis, and Effect of Remedies; Extirpation of the Uterus; Consequences of the Operation on the Sexual Condition of the Female Organs, after the Extirpation of the Uterus;* will not admit of analysis, being made up of transcriptions (without the "*rational deduction* which might settle the opinions and practice of obstetricians in the treatment of the disease," which the preface led us to expect,) from, perhaps, nearly all the writers on the subject. It appears, indeed, merely a *cento* copied from a common-place book. The author, however, apologises for this in the preface, but we consider his grounds for the construction of a book on this plan are not valid: compilations of this kind, if admissible in any form, should be confined to dictionaries and systems. The case certainly merits being generally known to the profession, but the advantages that may be derived from it would have equally ensued from its publication through the medium of some one of the periodical Medical Journals.

